# *N*-Substituted 2-Isonicotinoylhydrazinecarboxamides — New Antimycobacterial Active Molecules

**DOI:** 10.3390/molecules19043851

**Published:** 2014-03-28

**Authors:** Zuzana Rychtarčíková, Martin Krátký, Martin Gazvoda, Markéta Komlóová, Slovenko Polanc, Marijan Kočevar, Jiřina Stolaříková, Jarmila Vinšová

**Affiliations:** 1 Department of Inorganic and Organic Chemistry, Faculty of Pharmacy, Charles University, Heyrovského 1203, 500 05 Hradec Králové, Czech Republic; E-Mails: rychtarz@faf.cuni.cz (Z.R.); martin.kratky@faf.cuni.cz (M.K.); komlm3aa@faf.cuni.cz (M.K.); 2 Faculty of Chemistry and Chemical Technology, University of Ljubljana, Aškerčeva 5, SI-1000 Ljubljana, Slovenia; E-Mails: martin.gazvoda@fkkt.uni-lj.si (M.G.); marijan.kocevar@fkkt.uni-lj.si (M.K.); 3 Laboratory for Mycobacterial Diagnostics and Tuberculosis, Regional Institute of Public Health in Ostrava, Partyzánské náměstí 7, 702 00 Ostrava, Czech Republic; E-Mail: Jirina.Stolarikova@zu.cz

**Keywords:** InhA inhibition, *in silico* docking, *in vitro* antimycobacterial activity, 2-isonicotinoylhydrazinecarboxamide, 5-(pyridine-4-yl)-1,3,4-oxadiazol-2-amine, tuberculosis

## Abstract

This report presents a new modification of the isoniazid (INH) structure linked with different anilines via a carbonyl group obtained by two synthetic procedures and with *N*-substituted 5-(pyridine-4-yl)-1,3,4-oxadiazole-2-amines prepared by their cyclisation. All synthesised derivatives were characterised by IR, NMR, MS and elemental analyses and were evaluated *in vitro* for their antimycobacterial activity against *Mycobacterium tuberculosis* H_37_Rv, *Mycobacterium avium* 330/88, *Mycobacterium kansasii* 235/80 and one clinical isolated strain of *M. kansasii* 6509/96. 2-Isonicotinoyl-*N*-(4-octylphenyl)hydrazinecarboxamide displayed an *in vitro* efficacy comparable to that of INH for *M. tuberculosis* with minimum inhibitory concentrations (MICs) of 1–2 μM. Among the halogenated derivatives, the best anti-tuberculosis activity was found for 2-isonicotinoyl-*N*-(2,4,6-trichlorophenyl)hydrazinecarboxamide (MIC = 4 μM). *In silico* modelling on the enoyl-acyl carrier protein reductase InhA confirmed that longer alkyl substituents are advantageous for the interactions and affinity to InhA. Most of the hydrazinecarboxamides, especially those derived from 4-alkylanilines, exhibited significant activity against INH-resistant nontuberculous mycobacteria.

## 1. Introduction

Tuberculosis (TB) is one of the most dangerous infectious diseases known. Alarming WHO data [1] mainly concerning an increasing number of multidrug-resistant (MDR-TB) and extensively drug-resistant (XDR-TB) forms of TB, have prompted the development of new, potent, fast-acting anti-tuberculosis drugs. Although a new drug, diarylquinoline bedaquiline (TMC-207), has been approved recently, the treatment of multidrug-resistant TB and latent forms has still not been satisfactorily resolved. Treatment of the MDR-TB infection requires shortening the total duration of therapy, improving potency against resistant strains and reducing the total expenditure and toxicity [2].

Isonicotinic acid hydrazide (INH, **1**) represents a unique front-line anti-tuberculosis drug with a high specificity towards *Mycobacterium tuberculosis* (*Mtb.*). Its mechanism of action includes multiple effects on lipids, glycolysis, biomembranes, proteins and nucleic acid synthesis. In fact, **1** is a prodrug that has to be activated by a catalase/peroxidase (KatG) inside the mycobacterium to form an isonicotinoyl radical and/or stable oxidative products. KatG couples the isonicotinic acyl with NADH to form an isonicotinic acyl-NADH complex. This complex binds tightly to the enoyl-acyl carrier protein reductase (InhA), thereby blocking the natural enoyl-AcpM substrate and inhibiting the synthesis of mycolic acid, the main building block of the mycobacterial cell wall [3]. Among others, these facts are briefly summarised in the review of Vinšová *et al.* [4]. The discovery of isoniazid was a major milestone in the chemotherapy of tuberculosis, but the development of INH-resistance has hampered its wide usage and therapeutic potential. The need to overcome the resistance on INH, which has been found to correlate with five different genes *katG*, *inhA*, *ahpC*, *kasA* and *ndh* [5], led to a huge number of modifications, as summarised in several review articles [4,6,7,8].

We have already described a variety of synthetic modifications of INH linked with another active molecule through a methine bridge [9], by conjugation with an aniline group with electron-withdrawing substituents [10] and *via* the preparation of a new type of “double” active molecules based on the fluorinated hydrazides of a benzoic acid scaffold as an isoniazid isostere [11]. The current report is part of our on-going research and deals with the modification of the INH structure by introducing a carbonyl group (in place of previously described methine bridge [9,10]) as the moiety that connects INH to other molecules. Recently, the thiocarbonyl linker between the 2-(trifluoromethyl)aniline moiety and INH was shown to contribute to the superior activity of the modified molecule [12]. Furthermore, the conversion of INH to the corresponding 1,3,4-oxadiazoles resulted in compounds with improved lipophilicity and activity; some of these compounds also exhibited significant activity against INH-resistant strains [13]. Here, we focused on the activity of compounds that have an INH linked with different anilines *via* a carbonyl group and *N*-substituted-5-(pyridine-4-yl)-1,3,4-oxadiazole-2-amines prepared by their cyclisation.

## 2. Results and Discussion

### 2.1. Chemistry

**Scheme 1:**
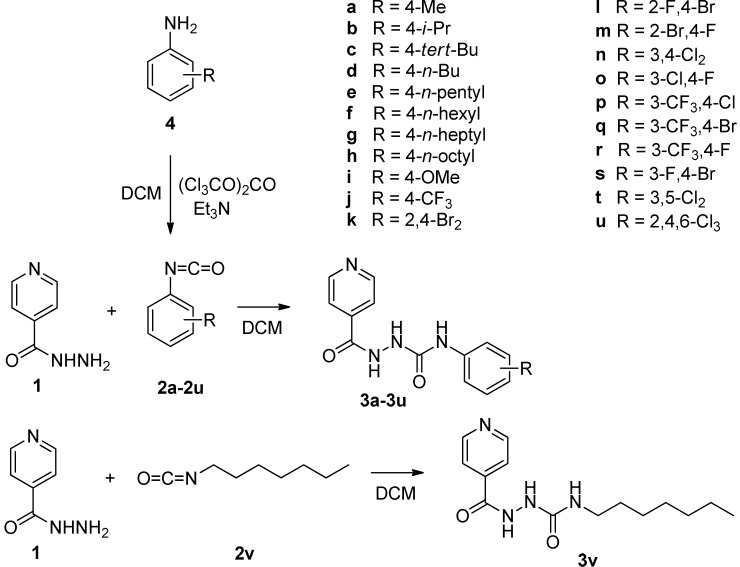
Synthesis of 2-isonicotinoyl-*N*-substituted-hydrazinecarboxamides **3**.

*Abbreviation*: DCM: dichloromethane.

The most straightforward method to obtain various *N*-substituted 2-isonicotinoylhydrazine-carboxamides **3** involves an addition of INH (**1**) to aryl isocyanates **2a**–**v**. Commercially available isocyanates were used for the synthesis of the 4-methyl, 4-isopropyl, 4-*tert*-butyl, 4-*n*-butyl and 4-methoxy derivatives **3a**–**d**, **3i** and *N*-heptyl derivative **3v** without a phenyl ring. To prepare the products **3e**–**h** and **3k**–**u**, the required aryl isocyanates **2e**–**h** and **2k**–**u** were generated *in situ* from the appropriate anilines **4** during treatment with triphosgene in the presence of triethylamine in dry dichloromethane (DCM; Scheme 1). These reaction intermediates were not isolated. The synthesis, subsequent isolation and purification gave yields within the range of 67%–97%.

**Scheme 2:**
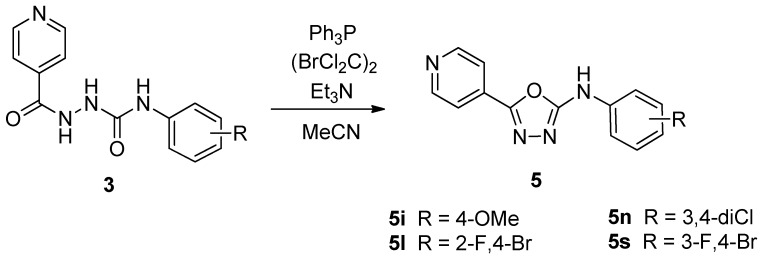
Synthesis of *N*-substituted-5-(pyridine-4-yl)-1,3,4-oxadiazol-2-amines **5**.

These described products **3** can serve as starting materials for the synthesis of 2,5-disubstituted 1,3,4-oxadiazoles. Namely, we previously developed an efficient procedure for the transformation of 1-acyl-4-substituted semicarbazides into the corresponding 1,3,4-oxadiazoles via *in situ* formed diazenes [14]. Some hydrazinecarboxamides **3**, because they belong to the same type of compounds mentioned above, were treated with the mixture of triphenylphosphine and 1,2-dibromo-1,1,2,2-tetrachloroethane in the presence of triethylamine in dry acetonitrile (MeCN) to generate the expected *N*-substituted-5-(pyridine-4-yl)-1,3,4-oxadiazol-2-amines **5**, as depicted in Scheme 2, with yields within the range of 33%–81%.

### 2.2. Antimycobacterial Activity

Compounds **3** and **5** were evaluated for their *in vitro* antimycobacterial activity against *M. tuberculosis* CNTC 331/88 (H_37_Rv), *M. avium* CNTC 330/88, *M. kansasii* CNTC 235/80 and *M. kansasii* 6509/96 (clinically isolated from a patient). The minimal inhibitory concentration (MIC) is the lowest concentration of the substance at which inhibition of the growth of *Mycobacterium* species occurs (Table 1).

None of the synthesised compounds of type **3** exhibited a higher activity than INH (MICs 0.5–1 μM) for *Mtb.* The best results were observed for the derivative **3h**, which reached an almost equal *in vitro* efficacy as isoniazid, with MICs of 1–2 μM. Among the halogenated molecules, the best activity was found for the 2,4,6-trichloro derivative **3u**, which had a MIC of 4 μM, followed by 2,4-dibromo and 3-CF_3_,4-bromo substituted molecules **3k** and **3q** (8 μM). These three derivatives also shared the highest calculated Clog*P* values (≥2.3) of all halogenated 2-isonicotinoyl-*N*-phenylhydrazinecarboxamides **3**.

When evaluating particular isomeric substitutions of the *N*-phenyl ring, we found that among the dichlorinated molecules, the 3,5-dichloro derivative **3t** showed a higher activity than the corresponding 3,4- derivative **3n** (32/62.5 μM and 125 μM), and for the bromofluoro derivatives, the MICs increased in the order 2-Br,4-F (**3m**; 16 μM) < 2-F,4-Br (**3l**; 32 μM) < 3-F,4-Br (**3s**; 62.5 μM). The comparison of the three molecules with a 3-CF_3_,4-halogen substitution led to the following result: the strongest activity against *Mtb.* was with the 3-CF_3_,4-Br derivative **3q** (8 μM), followed by 3-CF_3_,4-Cl **3p** and 3-CF_3_,4-F **3r** (32 μM).

When the *N*-(4-heptylphenyl) derivative **3g** and the *N*-heptyl molecule **3v**, which vary by the presence of a phenyl ring, were compared they exhibited identical MICs of 16/32 μM despite very different log*P* values. The influence of the 4-alkyl length on the activity is interesting: the expanding length from methyl **3a** to *n*-butyl **3d** increased the activity from 62.5 to 4/8 μM; pentyl **3e** and hexyl **3f** resulted in a milder activity; and heptyl **3g** displayed lower MICs comparable to the isopropyl. Finally, the octyl substituted molecule **3h** was evaluated and was determined to be the most potent compound in this series. One possible hypothesis that may explain this effect is the similarity of longer alkyl chain with fatty acids, which are structural fragments of biomembranes.

**Table 1:** Antimycobacterial activity of *N*-substituted 2-isonicotinoylhydrazinecarboxamides **3** and *N*-substituted 5-(pyridin-4-yl)-1,3,4-oxadiazol-2-amines **5**. 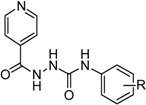

Code	R	MIC [μM]										
		*M. tuberculosis*		*M. avium*		*M. kansasii*			*M. kansasii*			C log*P*
		331/88		330/88		235/80			6509/96			
		14 d	21 d	14 d	21 d	7 d	14 d	21 d	7 d	14 d	21 d	
**3a**	4-methyl	62.5	62.5	**16**	**32**	62.5	125	>125	62.5	62.5	125	1.13
**3b**	4-isopropyl	16	32	**16**	**32**	**16**	**16**	**32**	**8**	**8**	**16**	1.87
**3c**	4-*tert*-butyl	8	16	62.5	125	**32**	62.5	125	32	62.5	62.5	2.34
**3d**	4-*n*-butyl	4	8	62.5	125	62.5	125	125	32	62.5	62.5	2.38
**3e**	4-pentyl	32	62.5	62.5	125	**32**	62.5	125	32	62.5	125	2.79
**3f**	4-hexyl	32	62.5	62.5	62.5	**32**	125	125	62.5	125	125	3.21
**3g**	4-heptyl	16	32	**32**	62.5	**32**	125	125	62.5	62.5	125	3.63
**3h**	4-octyl	**1**	**2**	250	500	250	500	>500	125	250	500	4.05
**3i**	4-OMe	62.5	62.5	**32**	62.5	>125	>125	>125	>125	>125	>125	0.51
**3j**	4-CF_3_	62.5	62.5	>125	>125	>125	>125	>125	125	125	125	1.56
**3k**	2,4-diBr	8	8	>125	>125	>125	>125	>125	>125	>125	>125	2.3
**3l**	2-F,4-Br	32	32	>125	>125	>125	>125	>125	125	>125	>125	1.63
**3m**	2-Br,4-F	16	16	>125	>125	>125	>125	>125	>125	>125	>125	1.63
**3n**	3,4-diCl	125	125	>125	>125	125	125	125	125	125	125	1.75
**3o**	3-Cl,4-F	62.5	125	>62.5	>125	>125	>125	>125	>125	>125	>125	1.35
**3p**	3-CF_3_,4-Cl	32	32	125	125	62.5	125	125	62.5	125	125	2.12
**3q**	3-CF_3_,4-Br	8	8	>125	>125	>125	>125	>125	125	125	125	2.39
**3r**	3-CF_3_,4-F	32	32	>125	>125	125	125	125	62.5	62.5	125	1.72
**3s**	3-F,4-Br	62.5	62.5	>125	>125	125	>125	>125	125	125	>125	1.63
**3t**	3,5-diCl	32	62.5	125	125	125	125	125	125	125	125	1.75
**3u**	2,4,6-triCl	4	4	>125	>125	>125	>125	>125	125	125	125	2.31
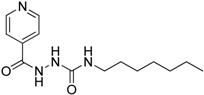												
**3v**	*-*	16	32	**32**	62.5	**32**	125	125	32	62.5	125	1.47
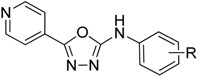												
**5i**	4-OMe	>125	>125	>125	>125	>125	>125	>125	>125	>125	>125	2.2
**5l**	2-F,4-Br	>125	>125	>125	>125	>125	>125	>125	>125	>125	>125	3.31
**5n**	3,4-diCl	>125	>125	>125	>125	>125	>125	>125	>125	>125	>125	3.44
**5s**	3-F,4-Br	>125	>125	>125	>125	>125	>125	>125	>125	>125	>125	3.31
**INH**		0.5	0.5-1	>250	>250	>250	>250	>250	4	8	8	−0.6

INH: isoniazid. The best MIC values for each strain are given in bold.

The situation for the atypical mycobacteria was different. Eleven hydrazinecarboxamides showed significantly lower MICs for *M. avium* than INH: **3a**–**g**, **3i**, **3p**, **3t** and **3v** (range, 16 to 125 μM) with the superiority of **3a** and **3b** (MICs of 16–32 μM) indicating that the best substitution pattern for the phenyl ring is a small 4-alkyl group (**3a**, **3b**) followed by 4-methoxy (**3i**), and 4-heptyl groups (**3g**). Identical MIC values were obtained for **3v**, the heptyl derivative without a phenyl ring. In general, *N*-substituted 2-isonicotinoyl hydrazinecarboxamides **3**, incorporating either alkyl (**3a**–**g**, **3v**) or methoxy (**3i**) groups, exhibited a significantly higher inhibition of *M. avium* growth than the halogenated derivatives **3j**–**u** (MICs ≥ 125 μM).

For both strains of *M. kansasii*, the alkyl substituted hydrazinecarboxamides **3a**–**g** and **3v** showed a higher *in vitro* potency than the halogenphenyl derivatives **3j**–**u**. Twelve compounds (**3a**–**g**, **3n**, **3p**, **3r**, **3t** and **3v**) inhibited INH-resistant strain 235/80, with MICs ≤ 125 μM, but no molecule produced lower MIC values than INH for clinical strain 6509/96. Only the 4-isopropyl derivative **3b**, the most active 2-isonicotinoylhydrazinecarboxamide **3** against all nontuberculous strains in this study (MICs 8–32 μM), displayed a similar *in vitro* efficacy. The most hydrophilic 4-methoxyphenyl **3i** and, surprisingly, the most lipophilic derivative 4-octylphenyl **3h** did not exhibit any significant activity. For the strain 6509/96, the presence of two or three chlorine atoms (**3n**, **3t**, **3u**) or a trifluoromethyl moiety at any position (**3j**, **3p**–**r**) on the phenyl ring led to better activity among the halogenated compounds. In general, there is no general and unequivocal correlation of Clog*P* and antimycobacterial activity within this series. This finding supports the conclusions of Ventura and Martins [15].

According to the interpretation of Scior and Garcés-Eisele [6], 2-isonicotinoylhydrazinecarbox-amides **3** act as INH (and then isonicotinic acid) prodrugs that must be activated. None of these compounds showed superiority over INH against drug-susceptible *M. tuberculosis* H_37_Rv. In contrast, a range of these compounds showed notable *in vitro* activity at micromolar concentrations against the INH-resistant strains of *M. avium* and *M. kansasii*. In the case of *M. avium*, due to the missing catalase/peroxidase enzyme, this observation can be explained on the basis of facilitated liberation of isonicotinoyl radicale from the less stable hydrazide derivative prodrugs [6].

An examination of the cyclised analogues of the four selected 2-isonicotinoylhydrazinecarboxamides with different substitution patterns (halogens: **3l**, **3n** and **3s**, and methoxy derivative **3i**), *i.e.*, *N*-substituted-5-(pyridine-4-yl)-1,3,4-oxadiazol-2-amines **5i**, **5l**, **5n** and **5s**, revealed a complete loss of efficacy (MICs > 125 μM; Table 1). Having these results in hand, we decided not to further study additional 5-(pyridine-4-yl)-1,3,4-oxadiazol-2-amines **5** as potential antimycobacterial agents.

In fact, the activity values for our 1,3,4-oxadiazol-2-amines are in sharp contrast with the results published by Navarrete-Vázques *et al.* [13] where the 2-position of the oxadiazole ring was occupied by alkyls, halogenoalkyls and aryls, and the MIC values were within the range of 0.35 to 49.69 μM for *Mtb.* H_37_Rv. Additionally, two other 2-substituted 5-(pyridine-4-yl)-1,3,4-oxadiazoles exhibited a remarkable inhibition of *M. tuberculosis* [16,17]. On the other hand, Foks *et al.* [18] reported substituted derivatives of 5-(pyridine-4-yl)-1,3,4-oxadiazol-2-amines, which avoid any significant activity against *Mtb*. 5-(Pyridine-4-yl)-1,3,4-oxadiazol-2-amine showed mild antimycobacterial activity; the replacement of the amino group by mercapto decreased the MIC [19]. The reason for the inactivity of *N*-(aryl substituted)-5-(pyridine-4-yl)-1,3,4-oxadiazole-2-amines **5** at the observed concentrations when compared to the previously reported derivatives [13,16,17] with other substituents at position 2, which are based on the hydrocarbon chains or rings, may originate from the changes in the hydrophilic-lipophilic balance due to the presence of an H-N bond as well as possible hydrogen bridge formations, different steric parameters or because these compounds may be metabolised by different ways within the cells.

### 2.3. Molecular Modelling Studies

The enoyl-acyl carrier protein reductase InhA, the target of INH, was further investigated as a possible target of INH derivatives. Molecular modelling studies were performed to suggest possible conformations of the novel compounds in the active site of the enzyme and to search for possible interactions between the ligand and InhA.

In previous studies [20,21], the crystal structure of InhA and its active site were described in detail. InhA belongs to the family of NADH – dependent dehydrogenase enzymes. Participating in fatty acid biosynthesis, the structure of InhA is adapted to accommodate its natural substrates, namely, long chained fatty acids. Adjacent to the NADH binding site is a substrate binding loop (residues 196–219) forming an oval-shaped cavity with plenty of hydrophobic amino acid residues. One side of the cavity is widely accessible to solvent. A crystallographic study of a ternary complex of InhA and C16 fatty acyl substrates revealed that the substrate occupies the substrate binding cavity in a U-shaped conformation, which is stabilised by surrounding hydrophobic side chains of amino acid residues at one side of the cavity and by hydrogen bonds with NADH at the other [22].

**Figure 1:**
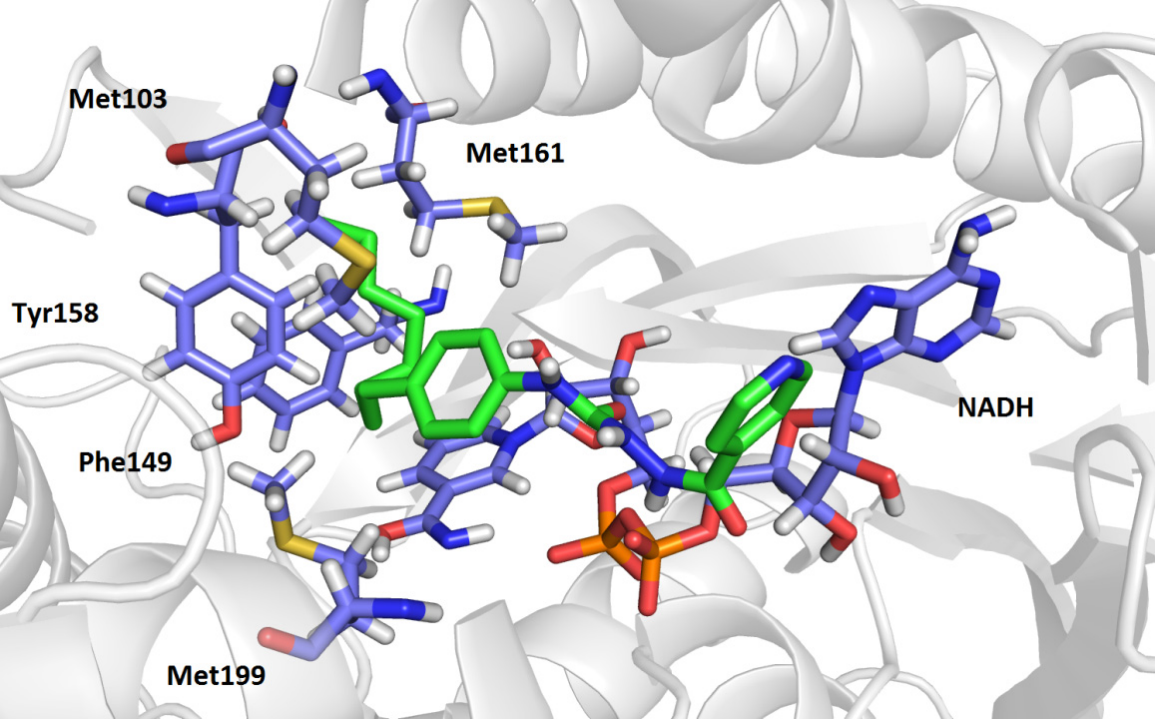
Conformation of compound 3h in the active site of InhA together with NAD and the interacting hydrophobic residues.

Molecular modelling studies of *N*-(4-alkylphenyl) substituted hydrazinecarboxamides (**3a**–**h**) indicate a similar binding of the derivatives to the active site of InhA, as in the previously described study of Rozwarski *et al.* [22]. The pyridine moiety of the ligands is positioned near the entrance to the cavity, and the hydrazinecarboxamide connecting linker is on the top of NADH within hydrogen bonding distance. Some of the molecules also display one direct hydrogen bond to the protein between the carboxamide nitrogen and the hydroxyl group of Tyr158. The lipophilic portion of the molecule point deep into the cavity and is surrounded by the hydrophobic side chains of Ile202, Leu218, Pro193, Met199, Met161, Met103, Phe149 and Ile215. The longer the alkyl substituent is, the more interactions are available in the area of the substrate cavity. One of the highest affinities towards the enzyme was observed in the case of 4-octylphenyl derivative **3h** (docking score of −8.6 kJ/mol; Figure 1).

Concerning the docking of variously halogenated derivatives, the conformation of these compounds was similar to that observed in the 4-alkylphenyl group with a pyridine moiety pointing towards the entrance of the cavity and a phenyl moiety pointing into the cavity. However, these compounds did not fill the substrate binding cavity as did the compounds with the longer alkyl substituents. This could result in the formation of fewer interactions with the hydrophobic residues and therefore lead to a weaker affinity towards the enzyme. The derivatives containing an oxadiazole moiety displayed similar conformation with hydrogen bonding to NADH and few hydrophobic interactions with Met103, Met199, Met161 and Phe149.

According to the observed conformations displayed by various derivatives, longer alkyl substituents are advantageous in the formation of interactions and therefore in the affinity to InhA. This phenomenon can be assumed from the structure of the natural substrates – long chain fatty acids. These results indicate that presented compounds **3** may affect InhA directly without previous necessary activation to form isonicotinoyl radical.

## 3. Experimental

### 3.1. General Information

The starting materials for the synthesis of the examined compounds were used as obtained from commercial sources (Sigma-Aldrich, Prague, Czech Republic, Fluka, Prague, Czech Republic, Penta Chemicals, Prague, Czech Republic, Fluka Alfa Aesar, Ljubljana, Slovenia, Maybridge Chemical Company Ltd, Ljubljana, Slovenia). The melting points were determined on a Kofler micro hot stage or on a Büchi Melting Point machine B-540 apparatus using open capillaries and were uncorrected. The NMR spectra were recorded at 29 °C on a Varian Mercury-Vxbb 300 (300 MHz for ^1^H and 75.5 MHz for ^13^C; Varian, Inc., Palo Alto, CA, USA) or with a Bruker Avance III 500 spectrometer (^1^H-NMR spectra at 500 MHz; ^13^C-NMR spectra at 125.8 MHz) using deuterated dimethyl sulfoxide (DMSO-*d*_6_) solutions of the samples. Proton spectra are referenced to TMS as the internal standard; the carbon shifts are given against the central line of the solvent signal (DMSO-*d*_6_ at *δ* = 39.5 ppm; CDCl_3_ at *δ* = 77.1 ppm). The coupling constants (*J*) are reported in Hz. IR spectra were obtained with a Perkin–Elmer Spectrum 100, equipped with a Specac Golden Gate Diamond ATR as a solid sample support, or they were recorded on a Nicolet 6700 FT-IR spectrometer in the range of 400–4,000 cm^−1^ (ATR). MS spectra were recorded with an Agilent 6224 Accurate Mass TOF LC/MS spectrometer. Elemental analyses (C, H, N) were performed with a Perkin Elmer 2400 Series II CHNS/O Analyzer. TLC was performed on Fluka silica-gel TLC-cards or on plates coated with 0.2 mm of Merck 60 F254 silica gel, and the chromatograms were visualised by UV irradiation (254 nm). The purity of synthesised compounds was checked by TLC, NMR, elemental analysis and for some compounds by HRMS. The calculated log*P* values (Clog*P*), which are the logarithms of the partition coefficients for octan-1-ol/water, were determined using the program CS ChemOffice Ultra version 12.0 (CambridgeSoft, Cambridge, MA, USA).

### 3.2. Chemistry

#### 3.2.1. Synthesis of Isonicotinoylhydrazinecarboxamides **3**

Method A

Isoniazid (**1**, 137.1 mg, 1.0 mmol) was suspended in anhydrous dichloromethane (5 mL), followed by the addition of the appropriate aryl isocyanate (**2**, 1.0 mmol) in one volume. The reaction mixture was stirred at room temperature for 30 min. A solid material was filtered off and recrystallised from boiling ethyl acetate. The reaction was monitored by TLC using the mixture *n*-hexane/ethyl acetate 3:1 as an eluent.

Method B

Triphosgene (120 mg, 0.4 mmol) was dissolved in anhydrous dichloromethane (5 mL) under argon atmosphere, and the appropriate aniline (**4**, 1.01 mmol) in dichloromethane (3 mL) was added dropwise. This mixture was stirred for 30 min at room temperature and then treated with triethylamine (293 µL, 2.1 mmol) in dichloromethane (3 mL). After 30 min, isoniazid (**1**, 138.5 mg, 1.01 mmol) was added. The reaction mixture was stirred for 30 min at room temperature, evaporated to dryness, treated with water (10 mL) and extracted with ethyl acetate (3 × 20 mL). The organic phase was dried over anhydrous sodium sulphate, filtered and evaporated to dryness to give the final product **3**, which was recrystallised from boiling ethyl acetate, if necessary. Some products were not sufficiently soluble to be extracted by cold ethyl acetate. In these cases, the organic layer of the suspension was filtered off, and the resulting crystals were recrystallised from boiling ethyl acetate and merged with the crystals obtained from the solution by evaporation of the solvent. The reaction was monitored by TLC using the mixture *n*-hexane/ethyl acetate 3:1 as an eluent.

*2-Isonicotinoyl-N-(4-methylphenyl)hydrazinecarboxamide* (**3a**). Method A; yield 97%; mp 227–229 °C; IR: 3273, 3030, 1673, 1640, 1591, 1516, 1487, 1407, 1339, 1310, 1288, 1234, 1090, 1065, 1020, 993, 918, 824, 775, 748, 683, 633 cm^−1^; ^1^H-NMR (500 MHz, DMSO-*d*_6_): *δ* 2.23 (3H, s), 7.07 (2H, d, *J* = 8.5 Hz), 7.35 (2H, d, *J* = 8.5 Hz), 7.82 and 8.78 (4H, AA’XX’), 8.25 (1H, bs), 8.80 (1H, bs), 10.58 (1H, bs); ^13^C-NMR (125.8 MHz, DMSO-*d*_6_): *δ* 20.3, 118.7, 121.4, 129.0, 130.8, 137.0, 139.6, 150.3, 155.4, 164.9; HRMS (ESI+) for C_14_H_15_N_4_O_2_ ([M+H]^+^): calcd. 271.1190; found 271.119. Anal. calcd. for C_14_H_14_N_4_O_2_ (270.29): C 62.21, H 5.22, N 20.73; found: C 61.97, H 4.95, N 20.59. 

*2-Isonicotinoyl-N-[4-(propan-2-yl)phenyl]hydrazinecarboxamide* (**3b**). Method A; yield 93%; mp 230–233 °C; IR: 3283, 3089, 2932, 1692, 1673, 1596, 1516, 1479, 1417, 1347, 1308, 1275, 1237, 1100, 1061, 1019, 1000, 971, 923, 883, 851, 827, 788, 757, 734, 698, 625, 604 cm^−1^; ^1^H-NMR (500 MHz, DMSO-*d*_6_): *δ* 1.18 (6H, d, *J* = 7.0 Hz), 2.82 (1H, sep, *J* = 7.0 Hz), 7.14 (2H, d, *J* = 8.5 Hz), 7.38 (2H, d, *J* = 8.5 Hz), 7.83 and 8.78 (4H, AA’XX’), 8.26 (1H, bs), 8.82 (1H, bs), 10.59 (1H, bs); ^13^C-NMR (125.8 MHz, DMSO-*d*_6_): *δ* 24.0, 32.8, 118.8, 121.4, 126.3, 137.2, 139.6, 142.0, 150.3, 155.4, 164.9; HRMS (ESI+) for C_16_H_19_N_4_O_2_ ([M+H]^+^): calcd. 299.1503; found 299.1512. Anal. calcd. for C_16_H_18_N_4_O_2_ (298.34): C 64.41, H 6.08, N 18.78; found: C 64.36, H 5.98, N 18.81. 

*2-Isonicotinoyl-N-[4-(tert-butyl)phenyl]hydrazinecarboxamide* (**3c**). Method A; yield 96%; mp 223.5–226 °C; IR: 3298, 3194, 3075, 2967, 2928, 1691, 1668, 1594, 1549, 1523, 1473, 1412, 1364, 1311, 1298, 1276, 1243, 1063, 1019, 1000, 920, 878, 844, 828, 754, 731, 705, 668 cm^−1^; ^1^H-NMR (300 MHz, DMSO-*d*_6_): *δ* 1.24 (9H, s), 7.26 (2H, d, *J* = 8.8 Hz), 7.37 (2H, d, *J* = 8.8 Hz), 7.81 and 8.76 (4H, AA’XX’), 8.25 (1H, bs), 8.83 (1H, bs), 10.58 (1H, bs); ^13^C-NMR (75.5 MHz, DMSO-*d*_6_): *δ* 31.5, 34.1, 118.7, 121.6, 125.4, 137.1, 139.8, 144.5, 150.5, 155.6, 165.1. Anal. calcd. for C_17_H_20_N_4_O_2_ (312.37): C 65.37, H 6.45, N 17.94; found: C 65.12, H 6.28, N 17.58. 

*2-Isonicotinoyl-N-(4-butylphenyl)hydrazinecarboxamide* (**3d**). Method A; yield 91%; mp 181–182.5 °C; IR: 3277, 3089, 2968, 2932, 2860, 1691, 1673, 1595, 1524, 1484, 1466, 1416, 1377, 1310, 1278, 1241, 1063, 1000, 923, 886, 850, 841, 823, 789, 753, 730, 701, 681, 669 cm^−1^; ^1^H-NMR (300 MHz, DMSO-*d*_6_): *δ* 0.87 (3H, t, *J* = 7.3 Hz), 1.27 (2H, sextet, *J* = 7.3 Hz), 1.50 (2H, p, *J* = 7.6 Hz), 2.49 (2H, t, *J* = 7.6 Hz), 7.06 (2H, d, *J* = 8.5 Hz), 7.35 (2H, d, *J* = 8.5 Hz), 7.81 and 8.76 (4H, AA’XX’), 8.80 (1H, bs), 8.24 (1H, bs), 10.57 (1H, bs); ^13^C-NMR (75.5 MHz, DMSO-*d*_6_): *δ* 14.0, 21.9, 33.5, 34.4, 119.0, 121.6, 128.6, 136.1, 137.4, 139.8, 150.5, 155.7, 165.1. Anal. calcd. for C_17_H_20_N_4_O_2_ (312.37): C 65.37, H 6.45, N 17.94; found: C 65.26, H 6.38, N 17.86. 

*2-Isonicotinoyl-N-(4-pentylphenyl)hydrazinecarboxamide* (**3e**). Method B; yield 81%; mp 195–196.5 °C; IR: 3277, 3049, 2955, 2922, 2855, 1673, 1637, 1611, 1589, 1562, 1536, 1489, 1467, 1408, 1378, 1341, 1304, 1283, 1241, 1068, 1052, 917, 845, 823, 787, 750, 722, 683, 668 cm^−1^; ^1^H-NMR (300 MHz, DMSO-*d*_6_): *δ* 0.84 (3H, t, *J* = 6.8 Hz), 1.34–1.18 (4H, m), 1.50 (2H, p, *J* = 7.4 Hz), 2.48 (2H, t, *J* = 7.5 Hz), 7.06 (2H, d, *J* = 8.4 Hz), 7.35 (2H, d, *J* = 8.4 Hz), 7.81 and 8.76 (4H, AA’XX’), 8.24 (1H, bs), 8.79 (1H, bs), 10.57 (1H, bs); ^13^C-NMR (75.5 MHz, DMSO-*d*_6_): *δ* 14.1, 22.1, 30.9, 31.0, 34.6, 119.0, 121.6, 128.5, 136.1, 137.4, 139.8, 150.5, 155.6, 165.1. Anal. calcd. for C_18_H_22_N_4_O_2_ (326.39): C 66.24, H 6.79, N 17.17; found: C 66.12, H 6.53, N 17.02. 

*2-Isonicotinoyl-N-(4-hexylphenyl)hydrazinecarboxamide* (**3f**). Method B; yield 84%; mp 177–179 °C; IR: 3271, 3082, 2960, 2929, 2856, 1691, 1672, 1597, 1516, 1482, 1416, 1378, 1340, 1310, 1279, 1239, 1063, 1000, 925, 889, 850, 815, 754, 700, 669 cm^−1^; ^1^H-NMR (300 MHz, DMSO-*d*_6_): *δ* 0.84 (3H, t, *J* = 7.0 Hz), 1.29–1.21 (6H, m), 1.51 (2H, p, *J* = 7.4 Hz), 2.48 (2H, t, *J* = 7.5 Hz), 7.06 (2H, d, *J* = 8.4 Hz), 7.35 (2H, d, *J* = 8.4 Hz), 7.81 and 8.76 (4H, AA’XX’), 8.25 (1H, bs), 8.80 (1H, bs), 10.58 (1H, bs); ^13^C-NMR (75.5 MHz, DMSO-*d*_6_): *δ* 14.2, 22.3, 28.5, 31.3, 31.3, 34.7, 119.0, 121.6, 128.6, 136.1, 137.4, 139.8, 150.5, 155.7, 165.1. Anal. calcd. for C_19_H_24_N_4_O_2_ (340.42): C 66.04, H 7.11, N 16.46; found: C 65.95, H 6.84, N 16.22.

*2-Isonicotinoyl-N-(4-heptylphenyl)hydrazinecarboxamide* (**3g**). Method B; yield 94%; mp 185.5–186.5 °C; IR: 3275, 3093, 2959, 2925, 2854, 1691, 1671, 1601, 1515, 1483, 1415, 1378, 1343, 1312, 1278, 1242, 1062, 1001, 924, 887, 851, 821, 782, 754, 733, 698, 670 cm^−1^; ^1^H-NMR (300 MHz, DMSO-*d*_6_): *δ* 0.83 (3H, t, *J* = 6.5 Hz), 1.32–1.18 (8H, m), 1.51 (2H, p, *J* = 7.2 Hz), 2.48 (2H, t, *J* = 7.2 Hz), 7.05 (2H, d, *J* = 8.1 Hz), 7.34 (2H, d, *J* = 8.1 Hz), 7.80 and 8.75 (4H, AA’XX’), 8.24 (1H, bs), 8.80 (1H, bs), 10.57 (1H, bs); ^13^C-NMR (75.5 MHz, DMSO-*d*_6_): *δ* 14.2, 22.3, 28.8, 28.8, 31.3, 31.5, 34.7, 119.0, 121.6, 128.6, 136.1, 137.4, 139.8, 150.5, 155.6, 165.1. Anal. calcd. for C_20_H_26_N_4_O_2_ (354.45): C 67.77, H 7.39, N 15.81; found: C 67.38, H 7.29, N 15.63.

*2-Isonicotinoyl-N-(4-octylphenyl)hydrazinecarboxamide* (**3h**). Method B; yield 93%; mp 189.5–190.5 °C; IR: 3276, 3194, 3087, 2922, 2854, 1691, 1671, 1594, 1519, 1479, 1417, 1379, 1340, 1310, 1278, 1241, 1062, 1000, 923, 887, 851, 825, 811, 751, 729, 696, 671, 655 cm^−1^; ^1^H-NMR (300 MHz, DMSO-*d*_6_): *δ* 0.84 (3H, t, *J* = 6.8 Hz), 1.31–1.18 (10H, m), 1.51 (2H, p, *J* = 6.7 Hz), 2.49 (2H, t, *J* = 7.4 Hz), 7.06 (2H, d, *J* = 8.4 Hz), 7.35 (2H, d, *J* = 8.4 Hz), 7.82 and 8.76 (4H, AA’XX’), 8.25 (1H, bs), 8.80 (1H, bs), 10.58 (1H, bs); ^13^C-NMR (75.5 MHz, DMSO-*d*_6_): *δ* 14.4, 22.5, 29.1, 29.1, 29.3, 31.6, 31.7, 34.9, 119.2, 121.9, 128.8, 136.4, 137.6, 140.1, 150.8, 155.9, 165.3. Anal. calcd. for C_21_H_28_N_4_O_2_ (368.47): C 68.45, H 7.66, N 15.21; found: C 68.23, H 7.50, N 15.06.

*2-Isonicotinoyl-N-(4-methoxyphenyl)hydrazinecarboxamide* (**3i**). Method A; yield 90%; mp 240–242 °C, IR: 3274, 3090, 2936, 1690, 1672, 1596, 1511, 1479, 1463, 1441, 1418, 1343, 1310, 1298, 1278, 1260, 1234, 1185, 1172, 1111, 1061, 1030, 999, 967, 922, 887, 850, 830, 808, 797, 770, 749, 698, 645, 627 cm^−1^; ^1^H-NMR (500 MHz, DMSO-*d*_6_): *δ* 3.71 (3H, s), 6.85 (2H, d, *J* = 8.5 Hz), 7.36 (2H, d, *J* = 8.5 Hz), 7.82 and 8.77 (4H, AA’XX‘), 8.22 (1H, bs), 8.73 (1H, bs), 10.57 (1H, bs); ^13^C-NMR (125.8 MHz, DMSO-*d*_6_): *δ* 55.1, 113.8, 120.5, 121.4, 132.5, 139.6, 150.3, 154.5, 155.6, 164.9; HRMS (ESI+) for C_14_H_15_N_4_O_3_ ([M+H]^+^): calcd. 287.1139; found 287.1139. Anal. calcd. for C_14_H_14_N_4_O_3_ (286.29): C 58.73, H 4.93, N 19.57; found C 58.48, H 4.63, N 19.53. 

*2-Isonicotinoyl-N-[4-(trifluoromethyl)phenyl]hydrazinecarboxamide* (**3j**). Method B; yield 67%; mp 207–210 °C; IR: 3115, 3029, 2170, 2113, 1755, 1731, 1674, 1634, 1610, 1554, 1498, 1451, 1428, 1335, 1267, 1221, 1110, 1083, 1066, 1015, 911, 879, 865, 840, 757, 726, 637, 613 cm^−1^; ^1^H-NMR (500 MHz, DMSO-*d*_6_): *δ* 7.62 (2H, d, *J* = 8.8 Hz), 7.70 (2H, d, *J* = 8.8 Hz), 7.82 and 8.79 (4H, AA’XX‘), 8.54 (1H, bs), 9.34 (1H, bs), 10.65 (1H, bs); ^13^C-NMR (125.8 MHz, DMSO-*d*_6_): *δ* 118.3 (q, *J* = 2.8 Hz), 121.4, 122.0 (q, *J* = 31.7 Hz), 124.5 (q, *J* = 271.1 Hz), 125.9 (q, *J* = 3.1 Hz), 139.5, 143.4, 150.4, 155.1, 164.9; HRMS (ESI+) for C_14_H_12_F_3_N_4_O_2 _([M+H]^+^): calcd. 325.0907; found 325.0916. Anal. calcd. for C_14_H_11_F_3_N_4_O_2_ (324.26): C 51.86, H 3.42, N 17.28; found C 51.73, H 3.25, N 17.10.

*N-(2,4-Dibromophenyl)-2-isonicotinoylhydrazinecarboxamide* (**3k**). Method B; yield 82%; mp 243–244 °C; IR: 3265, 1680, 1652, 1582, 1556, 1519, 1467, 1405, 1377, 1335, 1291, 1266, 1252, 1092, 1078, 1064, 1039, 919, 861, 834, 787, 751, 731, 683, 636 cm^−1^; ^1^H-NMR (500 MHz, DMSO-*d*_6_): *δ* 7.54 (1H, dd, *J*_1_ = 8.5 Hz, *J*_2_ = 1.3 Hz), 7.81 and 8.78 (4H, AA’XX‘), 7.86 (1H, d, *J* = 1.3 Hz), 7.96 (1H, d, *J* = 8.5 Hz), 8.32 (1H, bs), 9.14 (1H, bs), 10.75 (1H, bs); ^13^C-NMR (125.8 MHz, DMSO-*d*_6_): *δ* 114.8, 121.4, 123.3, 131.1, 134.2, 136.5, 139.3, 150.4, 154.8, 164.8; HRMS (ESI+) for C_13_H_11_Br_2_N_4_O_2 _[M+H]^+^): calcd. 412.9244; found 412.9255. Anal. calcd. for C_13_H_10_Br_2_N_4_O_2_ (414.05): C 37.71, H 2.43, N 13.53; found C 37.85, H 2.30, N 13.46.

*N-(4-Bromo-2-fluorophenyl)-2-isonicotinoylhydrazinecarboxamide* (**3l**). Method B; yield 96%; mp 235–238 °C; IR: 3298, 1645, 1597, 1554, 1528, 1489, 1408, 1329, 1306, 1248, 1195, 1122, 1093, 1069, 1050, 994, 891, 852, 814, 762, 751, 712, 687, 642 cm^−1^; ^1^H-NMR (500 MHz, DMSO-*d*_6_): *δ* 7.33–7.36 (1H, m), 7.55–7.59 (1H, m), 7.81 and 8.78 (4H, AA’XX‘), 7.87–8.02 (1H, m), 8.65 (1H, bs), 8.86 (1H, bs), 10.72 (1H, bs); ^13^C-NMR (125.8 MHz, DMSO-*d*_6_): *δ* 113.2, 118.5 (d, *J* = 22.6 Hz), 121.4, 122.2, 126.9 (d, *J* = 9.9 Hz), 127.5 (d, *J* = 3.0 Hz), 139.4, 150.4, 152.3 (d, *J* = 244.6 Hz), 154.8, 164.8; HRMS (ESI+) for C_13_H_11_BrFN_4_O_2_ ([M+H]^+^): calcd. 353.0044; found 353.0062. Anal. calcd. for C_13_H_10_BrFN_4_O_2_ (353.15): C 44.21, H 2.85, N 15.87; found C 44.09, H 2.80, N 15.71.

*N-(2-Bromo-4-fluorophenyl)-2-isonicotinoylhydrazinecarboxamide* (**3m**). Method B; yield 74%; mp 227–229 °C; IR: 3262, 3019, 1670, 1649, 1599, 1554, 1484, 1405, 1389, 1339, 1255, 1189, 1098, 1065, 1032, 923, 903, 854, 845, 814, 785, 750, 672 cm^−1^; ^1^H-NMR (500 MHz, DMSO-*d*_6_): *δ* 7.22–7.27 (1H, m), 7.60–7.63 (1H, m), 7.81 and 8.78 (4H, AA’XX‘), 7.87–7.90 (1H, m), 8.28 (1H, s), 8.96 (1H, bs), 10.72 (bs); ^13^C-NMR (125.8 MHz, DMSO-*d*_6_): *δ* 115.0 (d, *J* = 21.8 Hz), 119.1 (d, *J* = 25.5 Hz), 121.4, 123.9, 133.6 (d, *J* = 2.4 Hz), 139.4, 143.0, 150.4, 151.1, 154.1 (d, *J* = 258.5 Hz), 164.8; HRMS (ESI+) for C_13_H_11_BrFN_4_O_2_ ([M+H]^+^): calcd. 353.0044; found 353.0046. Anal. calcd. for C_13_H_10_BrFN_4_O_2_ (353.15): C 44.21, H 2.85, N 15.87; found C 44.19, H 2.69, N 15.75.

*N-(3,4-Dichlorophenyl)-2-isonicotinoylhydrazinecarboxamide* (**3n**). Method B; yield 68%; mp 220–222 °C; IR: 3287, 3081, 2932, 1692, 1666, 1588, 1515, 1472, 1395, 1328, 1295, 1263, 1227, 1129, 1064, 1030, 1000, 904, 865, 854, 845, 810, 784, 741, 709, 690, 676, 658, 620 cm^−1^; ^1^H-NMR (500 MHz, DMSO-*d*_6_): *δ* 7.45–7.47 (1H, m), 7.52 (1H, d, *J* = 8.5 Hz), 7.82 and 8.79 (4H, AA’XX‘), 7.88 (1H, d, *J* = 2.0 Hz), 8.59 (1H, bs), 9.25 (1H, bs), 10.64 (1H, bs); ^13^C-NMR (125.8 MHz, DMSO-*d*_6_): *δ* 118.7, 119.8, 121.4, 123.3, 130.5, 130.8, 139.5, 139.9, 150.4, 155.2, 164.9; HRMS (ESI+) for C_13_H_11_Cl_2_N_4_O_2 _([M+H]^+^): calcd. 325.0254; found 325.0254. Anal. calcd. for C_13_H_10_Cl_2_N_4_O_2_ (325.15): C 48.02, H 3.10, N 17.23; found C 47.75, H 2.95, N 17.05. 

*N-(3-Chloro-4-fluorophenyl)-2-isonicotinoylhydrazinecarboxamide* (**3o**). Method B; yield 83%; mp 212–214 °C; IR: 3311, 3183, 3098, 2932, 1689, 1665, 1602, 1539, 1499, 1482, 1406, 1329, 1304, 1287, 1261, 1246, 1209, 1135, 1095, 1066, 1054, 1001, 931, 910, 866, 852, 819, 805, 749, 700, 679, 661, 631, 611 cm^−1^; ^1^H-NMR (500 MHz, DMSO-*d*_6_): *δ* 7.30–7.34 (1H, m), 7.39–7.41 (1H, m), 7.77–7.80 (1H, m), 7.82 and 8.78 (4H, AA’XX‘), 8.52 (1H, bs), 9.12 (1H, bs), 10.61 (1H, bs); ^13^C-NMR (125.8 MHz, DMSO-*d*_6_): *δ* 116.7 (d, *J* = 21.6 Hz), 119.0 (d, *J* = 18.1 Hz), 120.0, 121.5, 136.9 (d, *J* = 2.8 Hz), 139.5, 149.6, 150.4, 152.4 (d, *J* = 241.2 Hz), 155.4, 165.0; HRMS (ESI+) for C_13_H_11_ClFN_4_O_2_ ([M+H]^+^): calcd. 309.0549; found 309.0549. Anal. calcd. for C_13_H_10_ClFN_4_O_2_ (308.70): C 50.58, H 3.27, N 18.15; found C 50.48, H 3.00, N 17.98. 

*N-[4-Chloro-3-(trifluoromethyl)phenyl]-2-isonicotinoylhydrazinecarboxamide* (**3p**). Method B; yield 91%; mp 231–234 °C; IR: 3294, 3188, 3081, 2936, 1692, 1668, 1601, 1515, 1479, 1407, 1319, 1301, 1279, 1259, 1221, 1184, 1127, 1064, 1033, 1008, 908, 889, 877, 846, 824, 804, 736, 696, 666, 609 cm^−1^; ^1^H-NMR (500 MHz, DMSO-*d*_6_): *δ* 7.61 (1H, d, *J* = 9.0 Hz), 7.78–7.84 (3H, m), 8.09 (1H, d, *J* = 2.5 Hz), 8.70 (1H, bs), 8.78 (2H, AA’XX‘), 9.40 (1H, bs), 10.66 (1H, bs); HRMS (ESI+) for C_14_H_11_ClF_3_N_4_O_2 _([M+H]^+^): calcd. 359.0517; found 359.0521. Anal. calcd. for C_14_H_10_ClF_3_N_4_O_2 _(358.70): C 46.88, H 2.81, N 15.62; found C 46.72, H 2.67, N 15.35.

*N-[4-Bromo-2-(trifluoromethyl)phenyl]-2-isonicotinoylhydrazinecarboxamide* (**3q**). Method B; yield 68%; mp 197–199 °C; IR: 3266, 1702, 1655, 1584, 1553, 1506, 1482, 1408, 1307, 1286, 1243, 1169, 1128, 1108, 1054, 998, 909, 890, 869, 842, 799, 760, 698, 681, 651 cm^−1^; ^1^H-NMR (500 MHz, DMSO-*d*_6_): *δ* 7.80–7.86 (5H, m), 8.31 (1H, bs), 8.79 (2H, AA’XX‘), 9.05 (1H, bs), 10.75 (1H, bs); HRMS (ESI+) for C_14_H_11_BrF_3_N_4_O_2_ ([M+H]^+^): calcd. 403.0012; found 403.0018. C_14_H_10_BrF_3_N_4_O_2_ (403.15). ¼ EtOAc: C 42.37, H 2.84, N 13.18; found C 42.72, H 3.17, N 13.44.

*N-[4-Fluoro-3-(trifluoromethyl)phenyl]-2-isonicotinoylhydrazinecarboxamide* (**3r**). Method B; yield 74%; mp 213–215 °C; IR: 3300, 3194, 3085, 2940, 1694, 1670, 1556, 1506, 1485, 1409, 1324, 1281, 1248, 1220, 1166, 1122, 1055, 1002, 911, 885, 850, 821, 749, 708, 653, 611 cm^−1^; ^1^H-NMR (500 MHz, DMSO-*d*_6_): *δ* 7.40–7.45 (1H, m), 7.74–7.81 (1H, m), 7.83 and 8.79 (4H, AA’XX‘), 7.96–7.99 (1H, m), 8.60 (1H, s), 9.28 (1H, bs), 10.63 (1H, bs); HRMS (ESI+) for C_14_H_11_F_4_N_4_O_2_ ([M+H]^+^): calcd. 343.0813; found 343.0829. Anal. calcd. for C_14_H_10_F_4_N_4_O_2_ (342.25): C 49.13, H 2.95, N 16.37; found C 48.97, H 2.72, N 16.03.

*N-(4-Bromo-3-fluorophenyl)-2-isonicotinoylhydrazinecarboxamide* (**3s**). Method B; yield 89%; mp 232–234 °C; IR: 3288, 3045, 1675, 1639, 1598, 1555, 1530, 1482, 1417, 1330, 1305, 1270, 1235, 1163, 1133, 1092, 1068, 1048, 993, 973, 912, 860, 846, 813, 772, 752, 711, 688, 650 cm^−1^. ^1^H-NMR (500 MHz, DMSO-*d*_6_): *δ* 7.22–7.25 (1H, m), 7.54–7.58 (1H, m), 7.64 (1H, dd, *J*_1_ = 11.5 Hz, *J*_2_ = 2.0 Hz), 7.81 and 8.78 (4H, AA’XX‘), 8.57 (1H, bs), 9.28 (1H, bs), 10.63 (1H, bs); ^13^C-NMR (125.8 MHz, DMSO-*d*_6_): *δ* 99.1 (d, *J* = 21.1 Hz), 106.6 (d, *J* = 27.2 Hz), 116.0, 121.4, 133.1, 139.5, 141.1 (d, *J* = 10.4 Hz), 150.4, 155.1, 158.1 (d, *J* = 241.7 Hz), 164.9; HRMS (ESI+) for C_13_H_11_BrFN_4_O_2_ ([M+H]^+^): calcd. 353.0044; found 353.0033. Anal. calcd. for C_13_H_10_BrFN_4_O_2_ (353.15): C 44.21, H 2.85, N 15.87; found 44.10, H 2.62, N 15.64. 

*N-(3,5-Dichlorophenyl)-2-isonicotinoylhydrazinecarboxamide* (**3t**). Method B; yield 74%; mp 226–229 °C; IR: 3491, 3228, 1682, 1586, 1535, 1471, 1448, 1413, 1344, 1296, 1248, 1226, 1111, 1067, 1021, 996, 928, 904, 841, 833, 802, 757, 704, 667, 624 cm^−1^; ^1^H-NMR (500 MHz, DMSO-*d*_6_): *δ* 7.17 (1H, t, *J* = 2.0 Hz), 7.61 (2H, bs), 7.82 and 8.79 (4H, AA’XX‘), 8.69 (1H, bs), 9.28 (1H, bs), 10.64 (1H, bs); ^13^C-NMR (125.8 MHz, DMSO-*d*_6_): *δ* 116.7, 121.1, 122.0, 134.0, 140.0, 142.2, 150.3, 155.1, 164.9; HRMS (ESI+) for C_13_H_11_Cl_2_N_4_O_2_ ([M+H]^+^): calcd. 325.0254; found 325.0254. Anal. calcd. for C_13_H_10_Cl_2_N_4_O_2_ (325.15): C 48.02, H 3.10, N 17.23; found C 47.76, H 2.80, N 16.92.

*2-Isonicotinoyl-N-(2,4,6-trichlorophenyl)hydrazinecarboxamide* (**3u**). Method B; yield 70%; mp 236–238 °C; IR: 3284, 1655, 1576, 1541, 1454, 1322, 1234, 853, 828, 745, 696, 635 cm^−1^; ^1^H-NMR (500 MHz, DMSO-*d*_6_): *δ* 7.73 (2H, s), 7.95 and 8.83 (4H, AA’XX‘), 8.69 (1H, bs), 8.73 (1H, bs), 10.78 (1H, bs); ^13^C-NMR (125.8 MHz, DMSO-*d*_6_): *δ* 122.3, 128.1, 131.7, 132.9, 135.2, 141.1, 148.9, 155.3, 164.3; HRMS (ESI+) for C_13_H_10_Cl_3_N_4_O_2_ ([M+H]^+^): calcd. 358.9864; found 358.9869. Anal. calcd. for C_13_H_9_Cl_3_N_4_O_2_ (359.60). 1/10 H_2_O: C 43.20, H 2.57, N 15.50; found C 42.45, H 2.49, N 15.15.

*N-Heptyl-2-isonicotinoylhydrazinecarboxamide* (**3v**). Method A; yield 90%; mp 150–152.5 °C; IR: 3290, 3214, 3096, 2924, 2855, 1671, 1636, 1554, 1522, 1508, 1486, 1436, 1411, 1375, 1339, 1296, 1221, 1142, 1063, 997, 904, 849, 758, 722, 677, 663 cm^−1^. ^1^H-NMR (300 MHz, DMSO-*d*_6_): *δ* 0.84 (3H, t, *J* = 6.9 Hz), 1.27–1.19 (8H, m), 1.38 (2H, p, *J* = 6.6 Hz), 3.00 (2H, q, *J* = 6.6 Hz), 6.54 (1H, t, *J* = 5.7 Hz), 7.78 and 8.73 (4H, AA’XX’), 7.91 (1H, bs), 10.38 (1H, bs). ^13^C-NMR (75 MHz, DMSO-*d*_6_): *δ* 14.2, 22.3, 26.5, 28.7, 30.0, 31.5, 40.5, 121.6, 140.0, 150.4, 158.2, 165.0. Anal. calcd. for C_14_H_22_N_4_O_2_ (278.35): C 60.41, H 7.97, N 20.13; found C 60.16, H 7.67, N 19.89. 

#### 3.2.2. General Procedure for the Synthesis of *N*-Substituted-1,3,4-oxadiazol-2-amines **5**

Triphenylphosphine (629.5 mg, 2.4 mmol) and 1,2-dibromo-1,1,2,2-tetrachloroethane (433.8 mg, 1.33 mmol) were added at room temperature to the stirred suspension of the appropriate isonicotinoylhydrazinecarboxamide (**3**, 1.2 mmol) in anhydrous acetonitrile (6 mL). The reaction mixture was stirred at room temperature for 5 min and cooled to 0 °C. Then, triethylamine (733 µL, 5.3 mmol) was added dropwise, and stirring was continued for an additional 30 min. A solid material was filtered off, washed with water (3 mL) and recrystallised from boiling methanol, giving the pure product **5**.

*N-(4-Methoxyphenyl)-5-(pyridin-4-yl)-1,3,4-oxadiazol-2-amine* (**5i**) [23]. Yield 48%; mp 233–235 °C (lit. 231.3–232.3 °C [23]); IR: 2788, 1644, 1598, 1573, 1508, 1439, 1416, 1305, 1253, 1238, 1215, 1176, 1037, 999, 967, 872, 835, 811, 794, 761, 719, 700, 680, 628 cm^−1^; ^1^H-NMR (500 MHz, DMSO-*d*_6_): *δ* 3.74 (3H, s), 6.97 (2H, d, *J* = 9.0 Hz), 7.54 (2H, d, *J* = 9.0 Hz), 7.79 and 8.78 (4H, AA’XX‘), 10.65 (1H, bs); ^13^C-NMR (125.8 MHz, DMSO-*d*_6_): *δ* 55.2, 114.4, 118.9, 119.1, 130.8, 131.5, 150.8, 154.7, 156.0, 160.8; HRMS (ESI+) for C_14_H_13_N_4_O_2_ ([M+H]^+^): calcd. 269.1033; found 269.1033. Anal. calcd. for C_14_H_12_N_4_O_2_ (268.27): C 62.68, H 4.51, N 20.88; found C 62.37, H 4.20, N 20.76.

*N-(4-Bromo-2-fluorophenyl)-5-(pyridin-4-yl)-1,3,4-oxadiazol-2-amine* (**5l**). Yield 81%, mp 262–264 °C; IR: 2940, 1634, 1610, 1568, 1491, 1412, 1309, 1199, 1128, 1054, 997, 886, 825, 700, 680 cm^−1^; ^1^H-NMR (500 MHz, DMSO-*d*_6_): *δ* 7.47–7.50 (1H, m), 7.63–7.67 (1H, m), 7.81 and 8.80 (4H, AA’XX‘), 8.08–8.13 (1H, m), 10.89 (1H, bs); ^13^C-NMR (125.8 MHz, DMSO-*d*_6_): *δ* 114.1 (d, *J* = 8.6 Hz), 118.9 (d, *J* = 22.0 Hz), 119.3, 122.0, 125.9 (d, *J* = 11.2 Hz), 127.8 (d, *J* = 3.5 Hz), 130.7, 150.8, 152.1 (d, *J* = 250.5 Hz), 156.9, 160.5; HRMS (ESI+) for C_13_H_9_BrFN_4_O ([M+H]^+^): calcd. 334.9938; found 334.9936. Anal. calcd. for C_13_H_8_BrFN_4_O (335.13): C 46.59, H 2.41, N 16.72; found C 46.50, H 2.29, N 16.64.

*N-(3,4-Dichlorophenyl)-5-(pyridin-4-yl)-1,3,4-oxadiazol-2-amine* (**5n**). Yield 33%; mp 257–259 °C; IR: 2766, 1635, 1589, 1567, 1475, 1404, 1302, 1249, 1129, 1051, 1001, 870, 819, 723, 703 cm^−1^; ^1^H-NMR (500 MHz, DMSO-*d*_6_): *δ* 7.54 (1H, dd, *J*_1_ = 8.8 Hz, *J*_2_ = 2.5 Hz), 7.62 (1H, d, *J* = 8.8 Hz), 7.79 and 8.79 (4H, AA’XX‘), 7.93 (1H, d, *J* = 2.5 Hz), 11.26 (1H, bs); ^13^C-NMR (125.8 MHz, DMSO-*d*_6_): *δ* 117.5, 118.4, 119.3, 123.6, 130.6, 131.0, 131.4, 138.4, 150.8, 156.5, 159.9; HRMS (ESI+) for C_13_H_9_Cl_2_N_4_O ([M+H]^+^): calcd. 307.0148; found 307.015. Anal. calcd. for C_13_H_8_Cl_2_N_4_O (307.13): C 50.84, H 2.63, N 18.24; found C 50.71, H 2.47, N 18.05.

*N-(4-Bromo-3-fluorophenyl)-5-(pyridin-4-yl)-1,3,4-oxadiazol-2-amine* (**5s**). Yield 77%; mp 260–262 °C; IR: 1642, 1592, 1566, 1487, 1417, 1255, 1174, 1054, 1174, 1054, 1002, 822, 750, 720, 703, 682, 658 cm^−1^; ^1^H-NMR (500 MHz, DMSO-*d*_6_): *δ* 7.35–7.38 (1H, m), 7.67–7.72 (2H, m), 7.82 and 8.79 (4H, AA’XX‘), 11.31 (1H, bs); ^13^C-NMR (125.8 MHz, DMSO-*d*_6_): *δ* 99.5 (d, *J* = 21.1 Hz), 105.4 (d, *J* = 27.6 Hz), 115.0 (d, *J* = 3.0 Hz), 119.3, 130.6, 133.8 (d, *J* = 1.2 Hz), 139.6 (d, *J* = 10.4 Hz), 150.9, 156.6, 158.4 (d, *J* = 242.8 Hz), 160.0; HRMS (ESI+) for C_13_H_9_BrFN_4_O ([M+H]^+^): calcd. 334.9938; found 334.9937. Anal. calcd. for C_13_H_8_BrFN_4_O (335.13). ⅕ H_2_O: C 46.09, H 2.50, N 16.54; found C 45.81, H 2.45, N 16.39. 

### 3.3. In Vitro Antimycobacterial Evaluation

The *in vitro* antimycobacterial activity of the prepared compounds was determined against *Mycobacterium tuberculosis* My 331/88 (H_37_Rv; dilution of strain, 10^−3^), *M. avium* My 330/88 (resistant to INH, rifampicin, ofloxacin and ethambutol; dilution of strain, 10^−5^), *M. kansasii* My 235/80 (dilution of strain, 10^−4^) and *M. kansasii* 6509/96 (dilution of strain, 10^−4^). All strains were obtained from the Czech National Collection of Type Cultures (CNCTC), with the exception of *M. kansasii* 6509/96, which was clinically isolated. The antimycobacterial activity of the compounds was determined in Šula’s semisynthetic medium (SEVAC, Prague, Czech Republic) via the micromethod for the determination of the minimum inhibitory concentration (MIC) at 37 °C after 14 and 21 days and after 7, 14 and 21 days for *M. kansasii* [24]. The tested compounds were added to the medium in DMSO solutions, and INH was used as a standard in a sterile water solution. The concentrations of the tested compounds were used as follows: 500, 250, 125, 62.5, 32, 16, 8, 4, 2, 1 and 0.5 μM. The same concentrations, over the range of 0.5 to 250 μM, were used for INH. The MIC (reported in μM) was the lowest concentration at which the complete inhibition of mycobacterial growth occurred.

### 3.4. Molecular Docking Studies

The crystal structure of InhA was obtained from the protein data bank (www.pdb.org, pdb code 1ENY), and its energy was minimised using UCSF Chimera software 1.6.2. [25]. The enzyme molecule was prepared using Autodock Tools 1.5.2. [26]. The hydrogens were added, partial charges were assigned and grid maps were generated. The ligand structure was created using CS ChemOffice version 10.0 (CambridgeSoft Cambridge, MA, USA), and its conformation was optimised with the aid of UCSF Chimera 1.6.2 using Amber force field.

Docking calculations were performed using Autodock 4.2.5.1. A three-dimensional affinity grid box was designed to include the full active site of InhA. The ligand molecule was kept fully flexible during the docking calculations, as well as some of the residues of the enzyme. The visualisations of the enzyme-ligand interactions were prepared using Pymol 1.1r1. [27].

## 4. Conclusions

The synthesis of new INH derivatives, 2-isonicotinoyl-*N*-(substituted)hydrazinecarboxamides, was performed from commercially available or *in situ* synthesised isocyanates with good yields, and their structures were confirmed by physico-chemical analyses. The biological test data for *Mycobacterium tuberculosis* showed that none of the synthesised compounds exhibited higher activity than INH, but the *N*-(4-octylphenyl) derivative **3h** reached almost the same *in vitro* efficacy with MICs 1-2 μM. Among the halogenated molecules, the best activity was found for the 2,4,6-trichloro derivative **3u**, with a MIC of 4 μM. An incorporation of the alkyl substituent on the carboxamide nitrogen of 2-isonicotinoylhydrazinecarboxamides led to a significantly higher inhibition of the growth of *M. avium* and both *M. kansasii* strains when compared to the halogenated derivatives and INH. Molecular modelling studies suggested a possible conformation of these novel compounds in the active site of the enzyme InhA. The 4-alkylphenyl group, the lipophilic portion, may be located in the cavity surrounded by hydrophobic side chains of amino acids; thus, the longer the alkyl substituent is, the more interactions are available in this area of the substrate cavity. Based on this hypothesis, derivatives bearing long alkyl or alkylphenyl connected with isoniazid moiety may bring an additional benefit.

Cyclisation of the selected compounds **3** to the corresponding 1,3,4-oxadiazol-2-amines **5** resulted in the loss of antimycobacterial efficacy. Although this observation is in contrast with previously published results, similar compounds of type **5** were not further investigated.
